# From Bonds to Barriers: Amphiphilic Molecules Enable Dual Interfacial and Electrolyte Regulation for Highly Stable Aqueous Zinc‐Ion Batteries

**DOI:** 10.1002/smll.74607

**Published:** 2026-07-16

**Authors:** Min Li, Chao Li, Lei Jiang, Xin Tan, Kwok‐Ho Lam, Qiaoji Zheng, Dunmin Lin

**Affiliations:** ^1^ College of Chemistry and Materials Science Sichuan Normal University Chengdu People's Republic of China; ^2^ Institute For Carbon Neutralization Technology College of Chemistry and Materials Engineering Wenzhou University Wenzhou Zhejiang People's Republic of China; ^3^ James Watt School of Engineering University of Glasgow Glasgow UK

**Keywords:** aqueous zinc‐ion batteries (AZIBs), hydrogen‐bond network, side reactions, Zn(101) crystal plane

## Abstract

The uncontrolled growth of zinc dendrites and severe parasitic side reactions critically impede the practical development of aqueous zinc‐ion batteries (AZIBs), largely due to the high activity of water molecules in aqueous electrolytes. Herein, we report arbutin (HQOGP) as a green and multifunctional electrolyte additive capable of simultaneously stabilizing the electrolyte and zinc anode interface. Comprehensive experimental investigations combined with theoretical calculations reveal that the hydrophilic glucose moiety of HQOGP reconstructs the hydrogen‐bonding network of the electrolyte, suppressing water activity and the hydrogen evolution reaction. Concurrently, the zincophilic hydroquinone group preferentially adsorbs onto the zinc surface, directing Zn^2+^ deposition along the thermodynamically favorable Zn(101) crystal plane and enabling uniform, compact, and dendrite‐free zinc growth. Benefiting from these synergistic effects, the Zn//Zn symmetric cell achieves stable cycling for 800 h at 10 mA cm^−2^, while the Zn//Cu half‐cell delivers highly reversible cycling over 1600 cycles at 1 mA cm^−^^2^. Furthermore, the Zn//VO_2_ full cell retains 87.73% of its initial capacity after 2000 cycles at 5 A g^−^^1^. This work demonstrates an effective strategy for concurrently mitigating dendrite formation and water‐induced side reactions through rational electrolyte design for durable aqueous metal batteries.

## Abstract 2

Amphiphilic HQOGP's glucose units suppress H_2_ evolution, hydroquinone groups guide Zn^2+^ to (101) plane for dense, dendrite‐free Zn, stabilizing anode.

## 1 Introduction

The rapid depletion of global fossil reserves and the escalating severity of environmental pollution have rendered the transition toward clean and sustainable energy systems inevitable [1, 2, 3]. In this context, secondary batteries play a pivotal role as core technologies for efficient energy storage, and breakthroughs in their performance are essential for reducing dependence on fossil fuels and enabling large‐scale integration of renewable energy sources such as wind and solar power [4, 5, 6]. Among various secondary battery technologies, lithium‐ion batteries (LIBs) have dominated the commercial market owing to their high energy density and excellent cycling stability [7, 8]. However, their further development is increasingly constrained by the limited abundance of lithium resources (0.0065%), high production cost, and intrinsic safety concerns associated with flammable organic electrolytes [9, 10].

In contrast, aqueous zinc‐ion batteries (AZIBs) have emerged as promising alternatives due to the natural abundance and low cost of zinc, whose theoretical specific capacity reaches 820 mAh g^−1^ [11]. Moreover, the two‐electron redox chemistry of Zn^2+^ enables higher energy output, while aqueous electrolytes offer inherent advantages such as non‐toxicity, non‐flammability, and environmental benignity, effectively eliminating the safety risks associated with organic electrolytes [12, 13, 14, 15, 16]. These merits position AZIBs as highly attractive candidates for large‐scale stationary energy storage and low‐speed electric vehicle applications [17]. However, the practical deployment of AZIBs remains hindered by the poor interfacial stability between the zinc metal anode and the aqueous electrolyte. Specifically, the uncontrollable growth of zinc dendrites can pierce the separator and induce internal short‐circuit, while parasitic hydrogen evolution reactions (HER) and subsequent surface passivation (e.g., formation of ZnO and Zn_4_SO_4_(OH)_6_·xH_2_O) continuously consume active zinc and electrolyte, resulting in rapid capacity decay and shortened battery lifespan [18, 19, 20, 21, 22, 23, 24, 25].

To address these challenges, extensive efforts have been devoted to stabilizing the zinc anode through strategies such as constructing artificial interface layers, designing three‐dimensional current collectors, and optimizing electrolyte compositions [26, 27, 28]. Among these approaches, the introduction of electrolyte additives has attracted considerable attention owing to its operational simplicity and high efficiency [29]. Traditional additive design mainly focuses on regulating the solvation structure of Zn^2+^ via coordination or hydrogen bonding, thereby forming a “water‐deficient” interfacial environment that suppresses side reactions [17, 30]. More recently, crystal plane regulation has emerged as a fundamentally important strategy, aiming to direct the deposition of zinc toward thermodynamically favorable crystal facets with optimized kinetic behavior through the selective adsorption of additive molecules [24, 31, 32, 33, 34]. This approach enables dense, uniform, and dendrite‐free zinc deposition.

Both theoretical calculations and experimental studies have demonstrated that among the various crystallographic planes of zinc, the (101) plane is particularly advantageous due to its relatively high surface energy and superior Zn^2+^ adsorption and diffusion kinetics [13, 35, 36]. For instance, cinnamaldehyde (CA) has been reported to function as a molecular surface regulator, inducing preferential deposition of Zn^2+^ along the (101) plane and enabling stable cycling of Zn//Cu half‐cells for 1000 cycles at a current density of 1 mA cm^−^^2^ [37]. Similarly, xylitol (Xyl) molecule selectively adsorbs onto the Zn(101) plane [38], triggering crystallographic reconstruction and facilitating horizontal epitaxial growth of zinc, which delivers stable cycling over 600 h at a high current density of 10 mA cm^−2^ and a capacity of 10 mAh cm^−2^. Such highly ordered deposition behavior markedly enhances the reversibility and cycling stability of zinc metal anodes.

**FIGURE 1:**
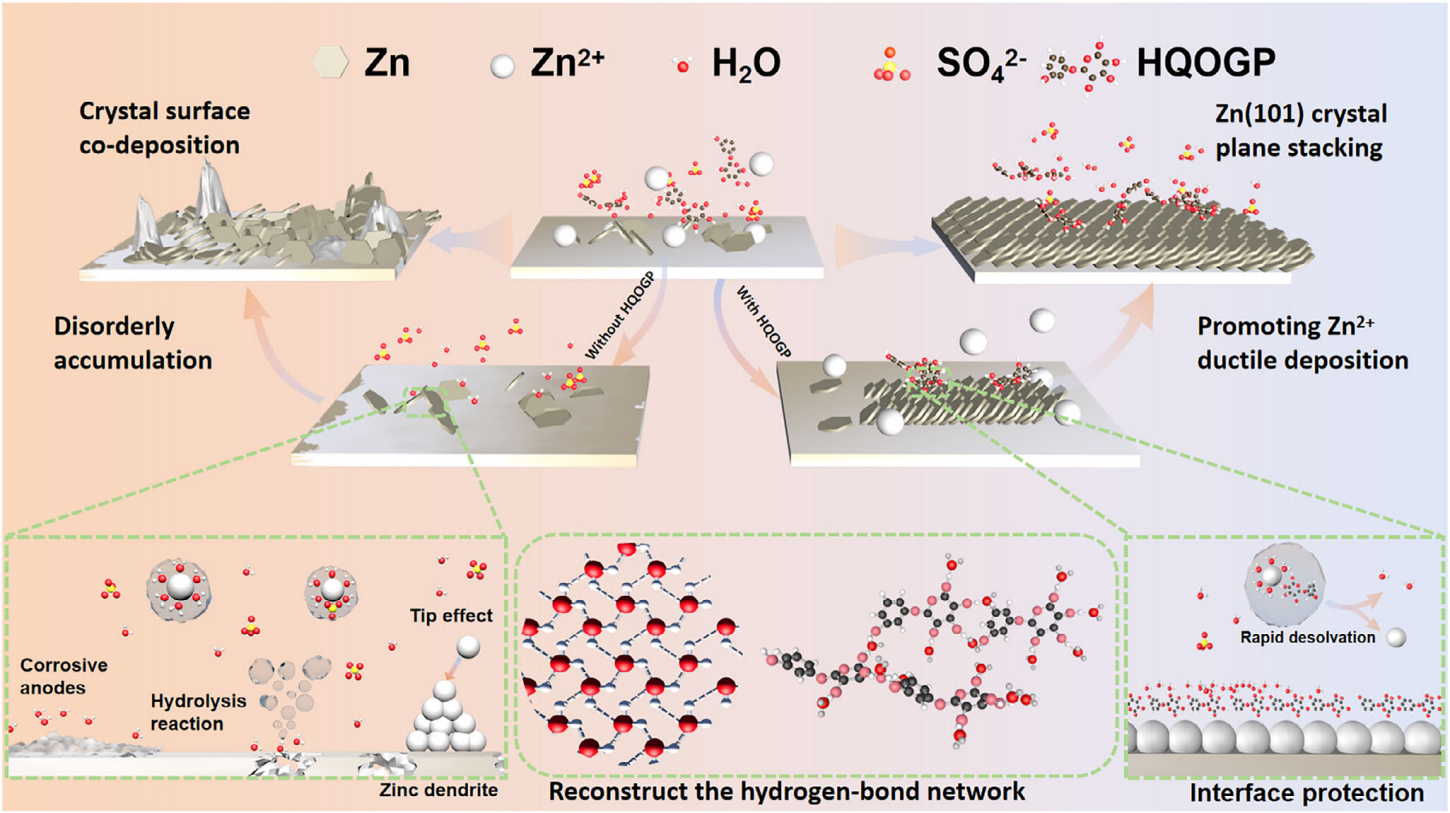
Schematic illustration of the zinc anode/electrolyte interfacial structure in pristine ZnSO_4_ and HQOGP‐containing electrolytes.

Inspired by these advances, we herein introduce arbutin (HQOGP), a natural derived, biodegradable, and commercially available compound, as a multifunctional electrolyte additive for AZIBs. Owing to its relatively low dosage (5 g L^−1^) and moderate commercial cost (∼US$52 kg^−1^), HQOGP offers a potentially practical approach for enhancing battery durability while maintaining reasonable electrolyte cost. The hydroquinone moiety of arbutin is designed to preferentially adsorb onto the zinc anode surface, thereby guiding uniform zinc deposition along the (101) crystal plane, constructing a water‐deficient interfacial layer, and suppressing HER‐induced corrosion. Meanwhile, the glucose unit of arbutin (hydroquinone O‐glycoside, HQOGP) effectively reconfigures the hydrogen‐bond network of the electrolyte and provides local pH buffering, further mitigating interfacial side reactions (Figure 1). As a result, the Zn//Zn symmetric cell exhibits stable cycling for over 1600 h at 2 mA cm^−2^ and 2 mAh cm^−2^. The Zn//Cu half‐cell achieves 1600 stable cycles at 1 mA cm^−2^ with an average Coulombic Efficiency (CE) of up to 99.7%, demonstrating highly reversible zinc plating/stripping behavior. Moreover, the Zn//VO_2_ full cell delivers a high specific capacity of 282.8 mAh g^−1^ and retains 87.73% of its initial capacity after 2000 cycles at 5 A g^−1^, highlighting the remarkable effectiveness of the arbutin‐based electrolyte in enhancing the performance and durability of AZIBs.

## 2 Experimental Section

### 2.1 Materials

All chemicals were used as received without further purification. Zinc sulfate heptahydrate (ZnSO_4_·7H_2_O, ≥99.5%, AR) was purchased from Chengdu Kolong Chemical Co. Ltd. Arbutin (HQOGP, 98%, AR) and vanadium pentoxide (V_2_O_5_, 99%) were obtained from Shanghai Maclean Biochemical Technology Co. Ltd. Deionized (DI) water was used throughout all experiments.

### 2.2 Preparation of Electrolytes

A baseline electrolyte was prepared by dissolving ZnSO_4_·7H_2_O in DI water to obtain a 2 m ZnSO_4_ solution. HQOGP was subsequently added to the 2 m ZnSO_4_ electrolyte at different concentrations (3.0, 5.0, and 10.0 g L^−1^) to form HQOGP‐containing electrolytes. Based on electrochemical performance optimization, the electrolyte containing 0.5 g L^−1^ HQOGP was identified as the optimal composition and used for subsequent studies unless otherwise specified.

### 2.3 Synthesis of VO_2_ Cathode

VO_2_ was synthesized via a one‐step hydrothermal method. Briefly, 1.0 g of V_2_O_5_ was dispersed in 40 mL of DI water, followed by the gradual addition of 10 mL of ethanol under continuous stirring. The mixture was stirred for an additional 10 min to ensure homogeneity and then transferred into a 100 mL Teflon‐lined stainless‐steel autoclave. The sealed autoclave was heated at 180°C for 24 h. After naturally cooling to room temperature, the resulting product was collected, washed three times with DI water and ethanol, and finally vacuum‐dried at 60°C for 24 h.

### 2.4 Zn//VO_2_ Battery Assembly

A CR2032 coin cells were assembled at room temperature using a 100 µm‐thick Zn foil as the anode and VO_2_ as the cathode. The VO_2_ mass loading was 0.6 mg cm^−2^, and electrolyte volume was 180 µL per cell. Glass fiber (Whatman, GF/a) was used as the separator. Based on the electrode capacities, the N/P ratio of the Zn//VO_2_ cell was calculated to be approximately 284.7.

### 2.5 Materials Characterizations and Electrochemical Measurements

X‐ray diffraction (XRD) patterns were recorded using an XRD diffractometer (Smart Lab, Rigaku, Japan) with Cu‐Kα radiation (λ = 1.540598 Å) over a 2*θ* range of 10°–80° at a scan rate of 8 min^−1^. The morphology of the samples was examined by field emission scanning electron microscopy (SEM, FEI, Sirion 200). Elemental composition and distribution were analyzed energy dispersive x‐ray spectroscopy (EDS) equipped on an SEM system.

CR2032‐type coin cells were assembled in ambient air, with 200 µL of electrolyte added to each cell. Zn//VO_2_ full cells were assembled using Zn foil (100 µm thickness) as the anode and synthesized VO_2_ as the cathode. Cyclic voltammetry (CV) measurements were performed in a three‐electrode configuration using a Ti plate as the working electrode, a Pt plate as the counter electrode, and an Ag/AgCl electrode as the reference electrode. Linear sweep voltammetry (LSV) and Tafel polarization measurements were conducted using a Zn plate as the working electrode, a Pt plate as the counter electrode, and an Ag/AgCl reference electrode. Chronoamperometry (CA) tests were carried out using a Zn plate as both the working and reference electrodes and a Pt plate as the counter electrode at a constant potential of −150 mV. Electrochemical impedance spectroscopy (EIS) measurements were performed over a frequency range from 100 kHz to 1 Hz. All electrochemical measurements were conducted using an electrochemical workstation (CHI760E, CHI660E, CH Instruments, Shanghai, China).

Zn//Cu half‐cells were assembled using Zn foil as the anode, 20 µm Cu foil as the cathode, and glass fiber as the separator. Coulombic efficiency (CE) tests were performed using a battery testing system (Land CT5001A, China). For in situ observation of Zn deposition behavior, a homemade transparent Zn//Zn cell was constructed, consisting of two Zn sheets fixed onto a glass substrate using transparent adhesive tape. The electrolyte was added to fully immerse the Zn electrodes. The transparent Zn//Zn cell was cycled at a current density of 10 mA cm^−2^ using a battery tester (LAND, China), while Zn dendrite growth was monitored and recorded in real time using a digital video microscope (NSZ‐808).

## 3 Results and Discussion

### 3.1 Effect of HQOGP on the Zinc Anode Interface

**FIGURE 2:**
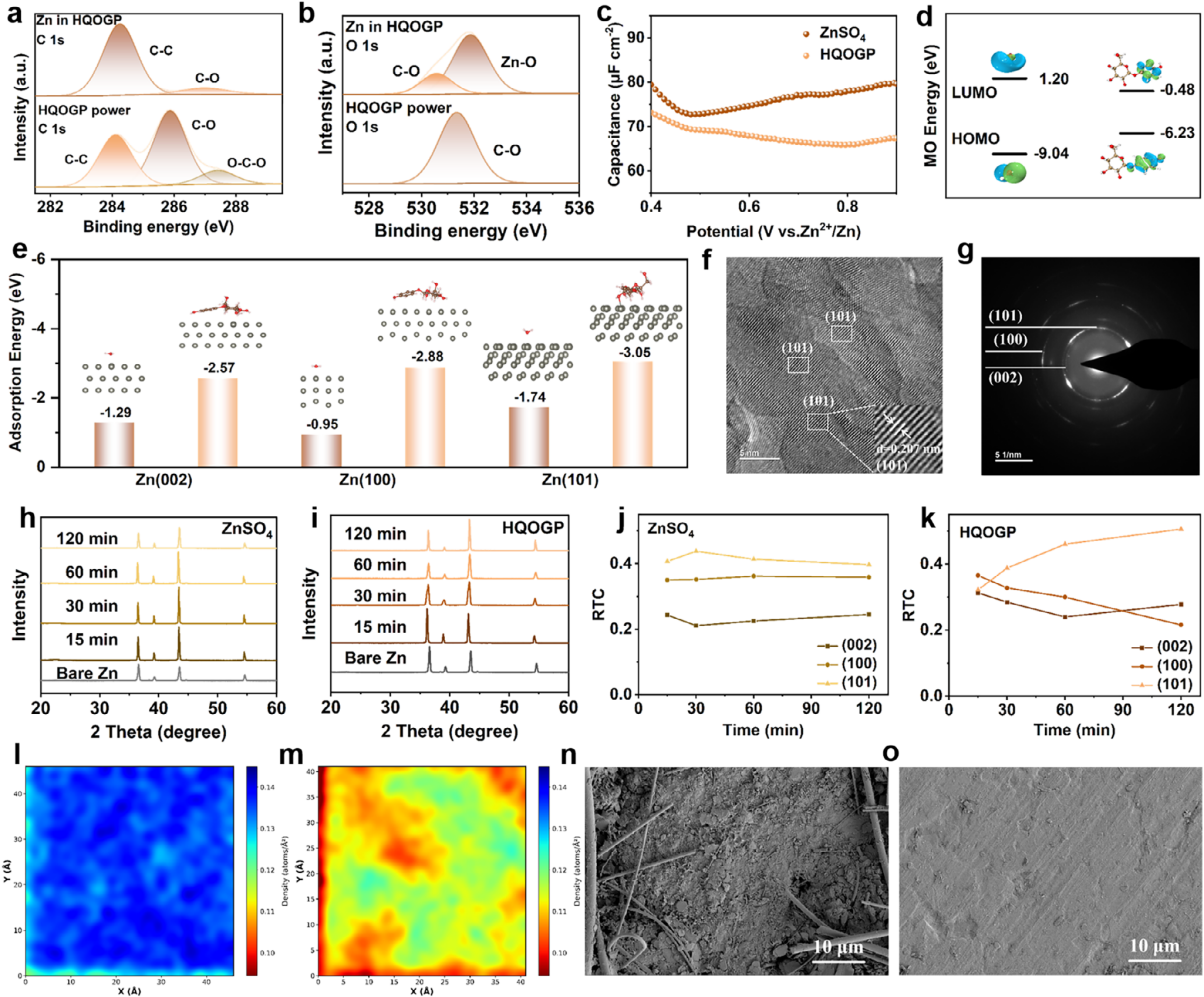
(a,b) XPS spectra of Zn foils after immersion in pristine ZnSO_4_ and HQOGP electrolytes. (c) Interfacial capacitance of Zn//Cu half‐cells measured in different electrolytes. (d) HOMO and LUMO isosurfaces of H_2_O (left) and HQOGP (right) molecules. (e) Calculated adsorption energies of H_2_O and HQOGP on different Zn crystallographic planes, including Zn(002), Zn(100), and Zn(101). (f) HRTEM image and (g) SAED pattern of deposited zinc. XRD patterns of zinc deposits formed in (h) pristine ZnSO_4_ and (i) HQOGP electrolytes at different deposition times. (j,k) RTC evolution of Zn(002), Zn(100), and Zn(101) planes obtained by fitting XRD data at different times. Molecular dynamics simulation results showing the spatial distribution of H_2_O molecules near the Zn anode in (l) pristine ZnSO_4_ and (m) HQOGP electrolytes. SEM image of zinc anodes after 50 cycles in symmetric cells operated in (n) pristine ZnSO_4_ and (o) HQOGP‐containing electrolytes.

To elucidate the interfacial regulation effect of HQOGP on the zinc anode, pristine zinc foils are immersed in a ZnSO_4_ electrolyte containing HQOGP for seven days prior to X‐ray photoelectron spectroscopy (XPS) characterization. As shown in Figure 2a, a distinct C–O characteristic peak emerges at 285.17 eV, indicating the successful involvement of HQOGP in modifying the interfacial chemical environment. In the O1s spectrum (Figure 2b), the presence of an O─Zn bonding peak at 531.88 eV further confirms the strong adsorption of HQOGP molecules onto the zinc surface.

Electrochemical double‐layer capacitance measurements (Figures S1 and S2) reveal a pronounced decrease in capacitance from 78.72 µF cm^−2^ in the pristine ZnSO_4_ electrolyte to 29.27 µF cm^−2^ upon HQOGP addition. The reduced capacitance cannot be solely attributed to a loss of electrochemically active area, as the HQOGP‐containing electrolyte simultaneously exhibits improved Zn plating/stripping reversibility, lower nucleation overpotential, and enhanced cycling stability. Instead, the decrease in capacitance is attributed to the preferential adsorption of HQOGP at the electrode/electrolyte interface, which modifies the interfacial charge distribution and partially screens the electric double layer. Consequently, HQOGP acts as a molecular shielding layer that provides interfacial protection while maintaining efficient charge‐transfer kinetics. This trend is consistent with the differential capacitance results (Figure 2c) and can be attributed to the preferential adsorption of HQOGP at the electrode–electrolyte interface. Molecular orbital calculations (Figure 2d) further show that the lowest unoccupied molecular orbital (LUMO) energy level of interfacial water (H_2_O) molecules (1.20 eV) is considerably higher than that of HQOGP (−0.48 eV), indicating superior electrochemical stability of HQOGP. Consequently, HQOGP preferentially adsorbs onto the zinc surface to form a stable protective layer, effectively reducing the local concentration of interfacial H_2_O molecules and suppressing water‐induced parasitic reactions.

To further clarify the adsorption behavior of HQOGP on different zinc facets, density function theory (DFT) calculations were conducted to evaluate the adsorption energies of H_2_O and HQOGP on the Zn(002), Zn(100), and Zn(101) planes. As summarized in Figure 2e, the adsorption energies of H_2_O on these planes (−1.29, −0.95, and −1.71 eV, respectively) are substantially less negative than those of HQOGP (−2.57, −2.88, and −3.05 eV). This marked difference indicates that HQOGP possesses a substantially stronger thermodynamic affinity for the zinc surface than water. Notably, the most negative adsorption energy is observed on the Zn(101) facet, which can be attributed to the aromatic π‐electron‐rich structure of the hydroquinone group, facilitating its strong preferential adsorption on this crystallographic plane. Notably, HQOGP exhibits the strongest adsorption on the Zn(101) plane, indicating its preferential interfacial anchoring and strong thermodynamic affinity for this facet. Such selective adsorption regulates the surface energy and growth kinetics of different crystallographic planes, stabilizing the Zn(101) facet during Zn nucleation and growth. As a result, uncontrolled deposition at high‐energy sites is suppressed, while the retention and growth of (101)‐oriented domains are promoted. This facet‐selective regulation ultimately directs Zn deposition along the Zn(101) orientation, leading to a more uniform and compact deposition morphology.

Transmission electron microscopy (TEM) analysis (Figure 2f) reveals numerous lattice fringes with an interplanar spacing of 0.207 nm, which closely matches the theoretical spacing of the Zn(101) plane. Correspondingly, selected‐area electron diffraction (SAED) patterns exhibit well‐defined diffraction spots indexed to the Zn(002), Zn(100), and Zn(101) planes (Figure 2g), corroborating the TEM observations. Furthermore, real‐time crystallography (RTC) results confirm that, compared to the pristine ZnSO_4_ electrolyte, the HQOGP‐containing system significantly promotes the preferential growth of the Zn(101) plane (Figure 2h–k and Figure S3), providing direct evidence that HQOGP regulates zinc deposition via crystallographic orientation control [39].

Chronoamperometry (CA) measurements (Figure S4) show that zinc deposition in the HQOGP electrolyte rapidly transitions from 2D to 3D diffusion within approximately 10 s, indicating accelerated and uniform nucleation kinetics. Consistently, nucleation overpotential measurements of Zn//Cu half‐cell (Figure S5) is 42 mV lower than that in the pristine ZnSO_4_ electrolyte, confirming that HQOGP facilitates dense and homogeneous Zn deposition. The preferential adsorption of HQOGP over H_2_O molecules leads to the formation of a water‐deficient interfacial layer. Interfacial H_2_O density analysis (Figure 2l,m) further demonstrates that the concentration of H_2_O molecules at the zinc surface is significantly reduced in the HQOGP system, effectively suppressing water‐related side reactions.

Scanning electron microscopy (SEM) images of zinc anodes after 50 cycles show severe surface roughening and heterogeneous deposits in the ZnSO_4_ electrolyte (Figure 2n). In sharp contrast, the zinc surface cycled in the HQOGP electrolyte exhibits a dense, smooth, and uniform morphology (Figure 2o). In situ optical microscopy observations (Figure S6) further confirm that HQOGP significantly enhances the uniformity of zinc deposition during cycling. Collectively, these results demonstrate that HQOGP preferentially adsorbs at the zinc anode interface, regulates Zn^2+^ deposition orientation, promotes uniform nucleation, and constructs a water‐poor protective layer, thereby effectively suppressing parasitic reactions and enhancing interfacial stability [40].

### 3.2 Effect of HQOGP on the Electrolyte

**FIGURE 3:**
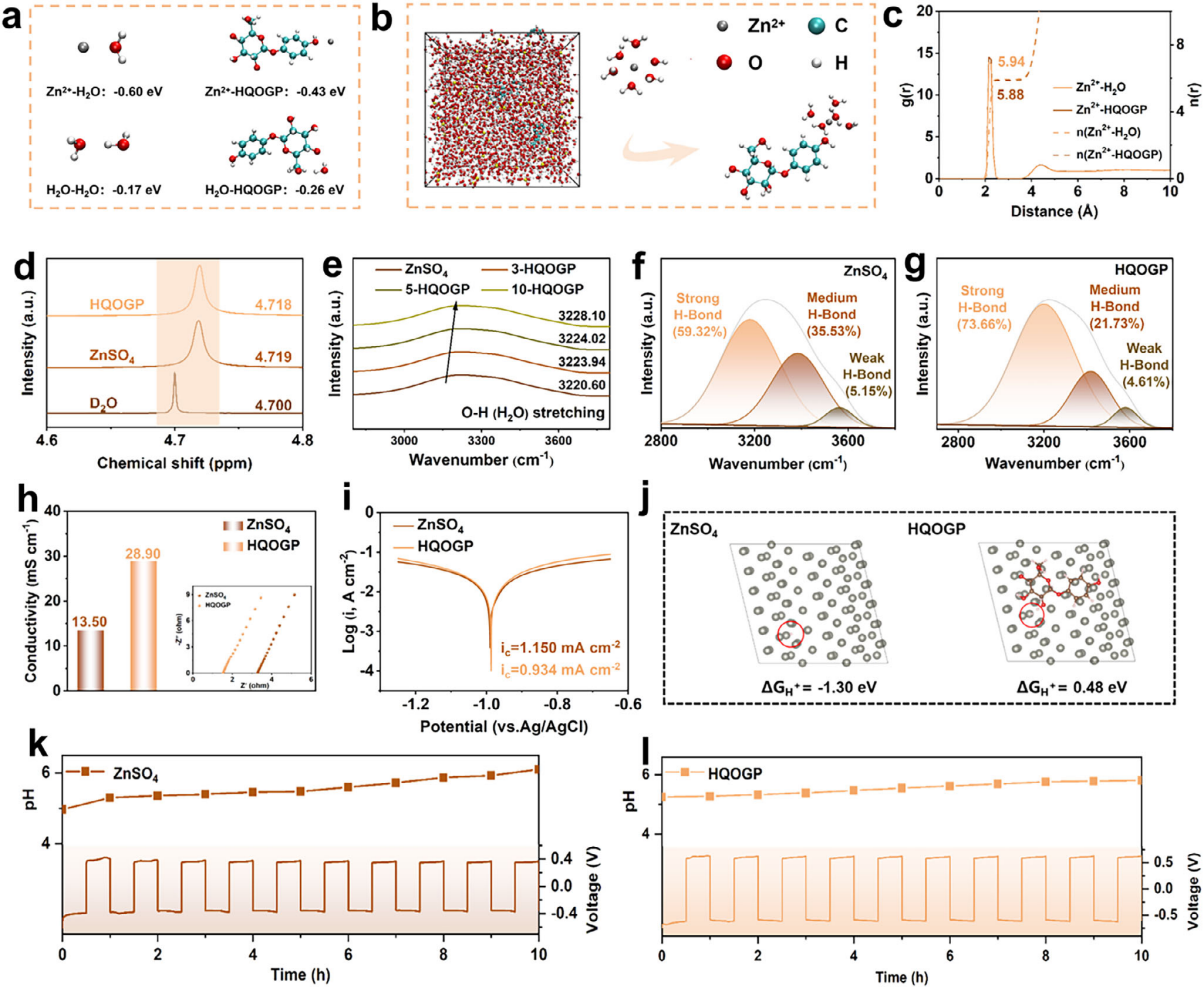
(a) Calculated binding energies of Zn^2+^–H_2_O, Zn^2+^–HQOGP, H_2_O–H_2_O, and H_2_O–HQOGP interactions. (b) Molecular dynamics (MD) simulation snapshots of ZnSO_4_ and HQOGP electrolytes. (c) RDFs and corresponding ACNs of Zn^2+^–O_water_. (d) ^1^H NMR spectra of electrolytes with and without HQOGP. (e) FTIR spectra highlighting O–H stretching vibrations in different electrolytes. Deconvoluted FTIR spectra of O─H stretching vibrations for (f) pristine ZnSO_4_ and (g) HQOGP electrolytes. (h) Ionic conductivity of Zn^2+^ in pristine ZnSO_4_ and HQOGP electrolytes. (i) Tafel polarization curves of Zn anodes in different electrolytes. (j) Comparison of Gibbs free energy for hydrogen adsorption (ΔG_H_^+^) on Zn anodes in ZnSO_4_ and HQOGP systems. Time–voltage profiles and in situ pH evolution near the Zn anode during plating/stripping in (k) pristine ZnSO_4_ and (l) HQOGP electrolytes.

To explore the influence of HQOGP on the electrolyte chemistry, the binding energies between Zn^2+^ and coordinating species were calculated (Figure 3a). The Zn^2+^–HQOGP binding energy (−0.43 eV) is less negative than that observed for Zn^2+^–H_2_O interactions in the pristine ZnSO_4_ electrolyte (−0.60 eV), indicating a relatively weak coordination between HQOGP and Zn^2^^+^. Molecular dynamics (MD) simulations (Figure 3b) reveal that in the [Zn(H_2_O)_5_(HQOGP)]^2+^ complex, HQOGP coordinates with Zn^2^^+^ via the hydroxyl group of the hydroquinone moiety. Radial distribution function (RDF) and coordination number (CN) analyses (Figure 3c) show that the addition of HQOGP reduces the number of H_2_O molecules in the first solvation shell of Zn^2+^ by only 0.06, suggesting that HQOGP exerts a minimal direct effect on the primary solvation structure of Zn^2+^.

^1^H NMR nuclear magnetic resonance (NMR) spectra (Figure 3d) exhibit a slight upfield shift of the water proton signal from 4.719 to 4.718 ppm upon HQOGP addition, indicating a moderate weakening of the strong Zn^2+^–H_2_O coordination and facilitating partial release of H_2_O molecules from the solvation sheath. Fourier transform infrared (FT‐IR) spectra (Figure 3e) show a gradual blue shift of the O─H stretching band (3000–3300 cm^−1^) with increasing HQOGP concentration, reflecting disruption and reconstruction of the hydrogen‐bond network in the electrolyte. Peak deconvolution of the O─H stretching region (Figure 3f,g) reveals that the fraction of strong hydrogen bonds increases from 59.32% in the pristine ZnSO_4_ electrolyte to 73.66% in the HQOGP‐containing electrolyte, accompanied by a corresponding decrease in medium and weak hydrogen bonds. This phenomenon originates from two simultaneous processes: disruption of the original H_2_O–H_2_O hydrogen‐bond by the insertion of HQOGP molecules and the formation of new, stronger HQOGP‐H_2_O hydrogen‐bond interactions. Notably, the glucose moiety of HQOGP, containing abundant hydroxyl groups, plays a dominant role in reconstructing the hydrogen‐bonding networks by forming extensive interactions with surrounding water molecules. This effectively reduces the activity of free water, thereby suppressing water‐induced side reactions. Consequently, a more stable hydrogen‐bond network is established in the presence of HQOGP.

Furthermore, the calculated binding energy between HQOGP and H_2_O molecules (−0.26 eV) is notably more negative than that between H_2_O molecules themselves (−0.17 eV) (Figure 3a), indicating that HQOGP stabilizes water clusters, reduces free water activity, and thereby suppresses water‐driven side reactions. Electrochemical impedance measurements show that the ion conductivity of the HQOGP electrolyte reaches 28.90 mS cm^−1^, substantially higher than that of the pristine ZnSO_4_ electrolyte (13.50 mS cm^−1^), demonstrating enhanced Zn^2+^ transport kinetics (Figure 3h). Tafel polarization curves (Figure 3i) reveal a reduced corrosion current density from 1.150 mA cm^−2^ in the ZnSO_4_ electrolyte to 0.934 mA cm^−2^ in the HQOGP system, indicating suppressed zinc corrosion. Furthermore, DFT calculations indicate that the Gibbs free energy of H^+^ adsorption (ΔG_H_^+^) on zinc decreases from −1.30 eV in the ZnSO_4_ electrolyte to 0.48 eV in the HQOGP system (Figure 3j), implying a significantly reduced thermodynamic driving force for the hydrogen evolution reaction.

During repeated charge–discharge cycling, the pH of the pristine ZnSO_4_ electrolyte increases sharply from 4.97 to 6.10 (Figure 3k), whereas the HQOGP electrolyte exhibits a more gradual pH change, rising from 5.25 to 5.81 (Figure 3l), confirming the effective buffering capability of HQOGP. During Zn plating/stripping, the HER generates OH^−^ ions at the zinc anode interface, resulting in local alkalization and an increase in electrolyte pH. In the pristine ZnSO_4_ electrolyte, the continuous accumulation of OH^−^ leads to a rapid pH rise. In contrast, the abundant hydroxyl groups of HQOGP can interact through hydrogen‐bonding and reversible proton‐transfer processes, thereby mitigating local OH^−^ accumulation and buffering pH fluctuations. Moreover, HQOGP suppresses HER through hydrogen‐bond network reconstruction, reducing OH^−^ generation at the source and resulting in a more gradual pH evolution during cycling. Collectively, these results demonstrate that HQOGP not only reconstructs the hydrogen‐bond network and stabilizes the electrolyte environment but also effectively suppresses HER and water‐related parasitic reactions, thereby markedly improving the interfacial stability of AZIBs.

### 3.3 Effect of HQOGP on the Electrochemical Performance of AZIBs

**FIGURE 4:**
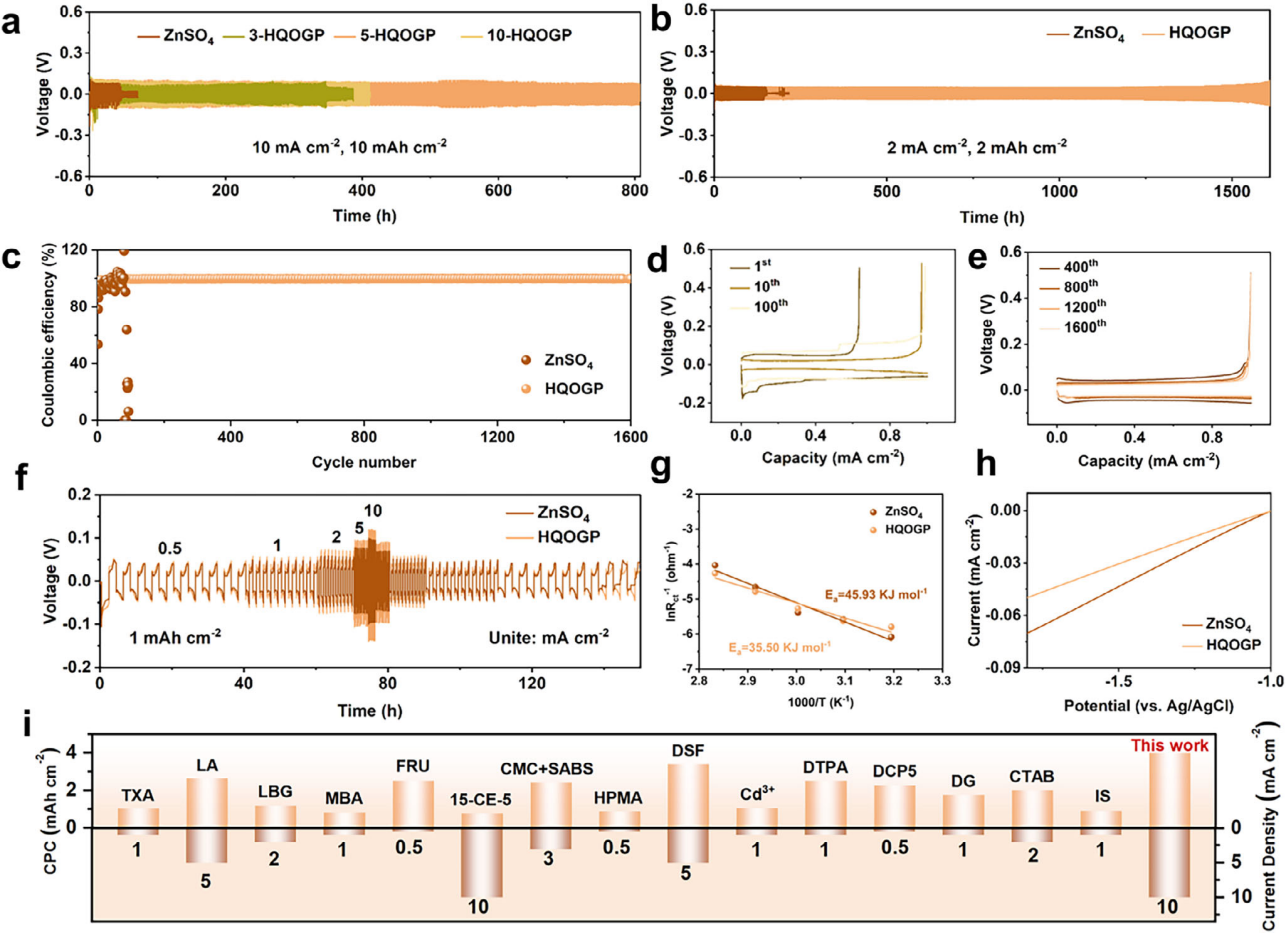
(a) Long‐term cycling performance of Zn//Zn symmetric cells at 10 mA cm^−2^ and 10 mAh cm^−2^. (b) Cycling stability of Zn//Zn symmetric cells at 2 mA cm^−2^ and 2 mAh cm^−2^. (c) CE of Zn//Cu half‐cells cycled at 1 mA cm^−2^ in 2 m ZnSO_4_ electrolytes with and without HQOGP. Corresponding voltage profiles of Zn//Cu half‐cells in (d) pristine ZnSO_4_ and (e) HQOGP electrolytes at selected cycles. (f) Zn plating/stripping voltage profiles of Zn//Zn symmetric cells in pristine ZnSO_4_ and HQOGP electrolytes at current densities ranging from 0.5 to 10 mA cm^−2^. (g) Arrhenius plots derived from temperature‐dependent impedance measurements of Zn//Zn symmetric cells in different electrolytes. (h) LSV curves of Zn anode in ZnSO_4_ and HQOGP electrolytes. (i) Comparison of cumulative plating capacity and cycle life of Zn//Zn symmetric cells using HQOGP electrolyte with previously reported systems.

To further evaluate the effectiveness of HQOGP as an electrolyte additive, Zn//Zn symmetric cells were assembled using ZnSO_4_ electrolytes containing different concentrations of HQOGP. Long‐term galvanostatic cycling tests were conducted under a current density of 10 mA cm^−2^. As shown in Figure 4a, the electrolyte containing 5 g L^−1^ of HQOGP exhibits the most stable cycling behavior, sustaining operation for over 800 h, significantly outperforming electrolytes with lower or higher concentrations. Consequently, 5 g L^−1^ of HQOGP was identified as the optimal additive concentration for subsequent experiments.

At a lower current density of 2 mA cm^−2^ and a real capacity of 2 mAh cm^−2^, the Zn//Zn symmetric cell employing pristine ZnSO_4_ electrolyte fails after only 142 h. In contrast, the cell using the HQOGP‐modified electrolyte delivers remarkably prolonged cycling stability exceeding 1600 h (Figure 4b), clearly demonstrating the effectiveness of HQOGP in stabilizing the zinc anode interface.

To further assess Zn plating/stripping reversibility, Zn//Cu half‐cells were assembled and cycled at 1 mA cm^−2^ (Figure 4c–e). In the pristine ZnSO_4_ electrolyte, the cell suffers from an internal short circuit after fewer than 100 cycles, primarily due to incomplete zinc stripping on the copper substrate, leading to the accumulation of electrochemically inactive “dead zinc” and accelerated dendrite growth. By contrast, the HQOGP‐containing electrolyte enables highly reversible Zn deposition/stripping, maintaining an average CE of 99.7% over 1600 cycles, underscoring the pronounced improvement in interfacial reversibility afforded by HQOGP.

The rate performance of Zn//Zn symmetric cells was systematically investigated over a current density range from 0.5 to 10 mA cm^−2^ (Figure 4f). Compared with the pristine ZnSO_4_ electrolyte, the HQOGP electrolyte exhibits a slightly higher polarization voltage (Figure S7), which is attributed to the strong adsorption of HQOGP molecules at the zinc/electrolyte interface. This controlled interfacial adsorption, although increasing polarization, effectively promotes uniform Zn^2+^ deposition and significantly enhances cycling stability and electrochemical reversibility. Exchange current densities were extracted from linear fitting of overpotential versus current density, revealing a reduction from 1.0964 mA cm^−2^ in the pristine system to 0.7537 mA cm^−2^ in the HQOGP electrolyte (Figure S8). Given that exchange current density is closely related to hydrogen concentration and surface reaction kinetics, this reduction indicates effective suppression of the hydrogen evolution reaction (HER). Linear sweep voltammetry (LSV) measurements further support this conclusion, as evidenced by a positively shifted HER onset potential in the HQOGP electrolyte (Figure 4h).

The Zn^2+^ transference number (*t*_Zn_^2+^) was determined using combined chronoamperometry and electrochemical impedance spectroscopy. The pristine ZnSO_4_ electrolyte exhibits a *t*_Zn_^2+^ value of 0.28 (Figure S9), whereas the HQOGP electrolyte achieves a substantially higher value of 0.61 (Figure S10), indicating significantly enhanced Zn^2+^ migration kinetics. Temperature‐dependent impedance measurements of Zn//Zn symmetric cells (Figure S11) were further analyzed using the Arrhenius relationship to extract the activation energy (*E*_a_) for ion transport. The calculated *E*_a_ decreases from 45.93 kJ mol^−1^ for the ZnSO_4_ electrolyte to 36.50 kJ mol^−1^ for the HQOGP electrolyte (Figure 4g), suggesting that HQOGP facilitates the formation of energetically favorable pathways for Zn^2+^ transport and accelerates interfacial charge transfer. Moreover, SEM images of zinc electrodes immersed in the two electrolytes for 7 days (Figures S12 and S13) confirm that HQOGP effectively mitigates interfacial parasitic reactions and surface corrosion. A comparative analysis of cumulative plating capacity (CPC) with previously reported electrolyte additives (Figure 4i and Table S1) demonstrates that the HQOGP system delivers superior cycling stability [17, 21, 41, 42, 43, 44, 45, 46, 47, 48, 49, 50, 51, 52], further validating its effectiveness in extending the service life of AZIBs.

### 3.4 Effect of HQOGP on the Performance of Zn//VO_2_ Full Cells

**FIGURE 5:**
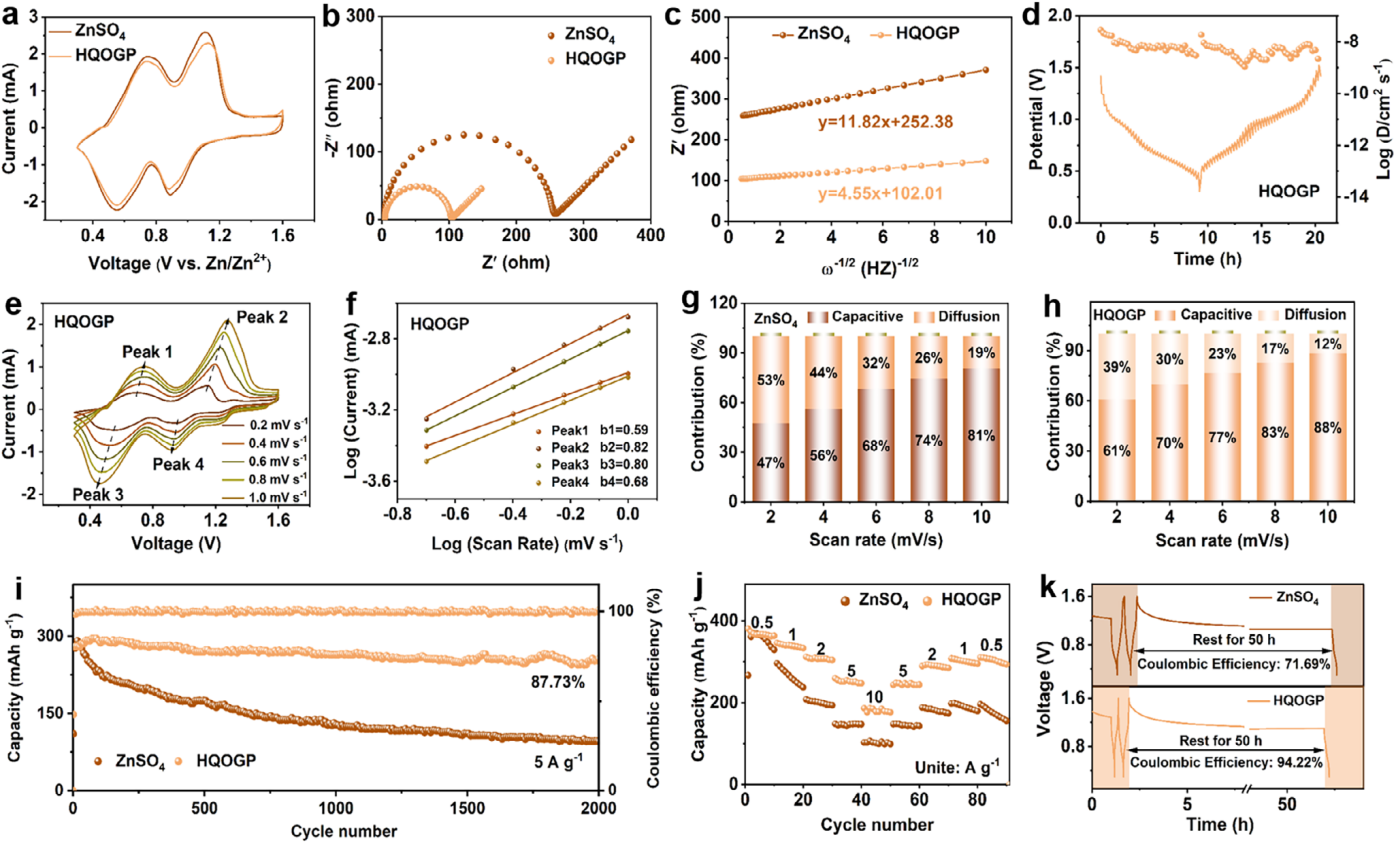
(a) CV curves of Zn//VO_2_ full cells recorded at 0.1 mV s^−1^ over a voltage range of 0.3–1.6 V. (b) EIS spectra of Zn//VO_2_ full cells in different electrolytes. (c) Linear fitting of *Z*′ versus *ω* ^−1/2^ for Zn//VO_2_ cells in pristine ZnSO_4_ and HQOGP electrolytes. (d) GITT profiles of Zn//VO_2_ cells in HQOGP electrolyte. Comparison of capacitive contribution to Zn^2^^+^ storage in Zn//VO_2_ cells using (e) pristine ZnSO_4_ and (f) HQOGP electrolytes. (g) CV curves of Zn//VO_2_ full cells recorded at scan rates of 0.2–1.0 mV s^−1^ in HQOGP electrolyte. (h) Corresponding log(*i*)–log(*v*) plots used to determine charge‐storage kinetics. (i) Long‐term cycling performance of Zn//VO_2_ full cells at 5 A g^−1^. (j) Rate capability of Zn//VO_2_ full cells in pristine ZnSO_4_ and HQOGP electrolytes. (k) Self‐discharge behavior of Zn//VO_2_ full cells after open‐circuit rest.

To demonstrate the practical applicability of the HQOGP electrolyte, Zn//VO_2_ full cells were assembled and systematically evaluated. X‐ray diffraction (XRD, PDF#31‐1438) (Figure S14) and SEM analyses (Figure S15) confirm the successful synthesis of the VO_2_ cathode material. Cyclic voltammetry (CV) curves of Zn//VO_2_ full cells with different electrolytes exhibit two well‐defined and overlapping redox peak pairs (Figure 5a), indicating that the introduction of HQOGP has a negligible effect on the intrinsic redox behavior of the VO_2_ cathode. Notably, electrochemical impedance spectroscopy (EIS) reveals a significantly lower charge‐transfer resistance for the HQOGP‐based cell compared to the pristine ZnSO_4_ system (Figure 5b), suggesting enhanced conductivity of Zn^2^^+^ and faster interfacial charge‐transfer kinetics.

Linear fitting of the real impedance (*Z*′) versus *ω* ^−12^ yields slopes of 11.82 and 4.55 for the ZnSO_4_ and HQOGP electrolytes, respectively (Figure 5c), indicating a markedly reduced diffusion resistance in the HQOGP system. Galvanostatic intermittent titration technique (GITT) measurements (Figure 5d and Figure S16) provide further evidence that the diffusion coefficient of Zn^2^^+^ at the VO_2_/electrolyte interface in the HQOGP electrolyte is approximately one order of magnitude higher than that in the pristine ZnSO_4_ electrolyte.

To gain deeper insight into the electrochemical kinetics, CV measurements were performed at scan rates ranging from 0.2 to 1.0 mV s^−1^ (Figure 5e and Figure S17). The CV curves maintain well‐defined and symmetrical shapes with increasing scan rate, reflecting highly reversible Zn^2^^+^ insertion/extraction processes. The relationship between peak current (i) and scan rate (v) was analyzed using the power‐law equation *i* = a·*v* ^b^. The extracted b values (Figure 5f and Figure S18) indicate that Zn^2+^ storage in the HQOGP electrolyte is predominantly governed by pseudocapacitive behavior, whereas the ZnSO_4_ electrolyte is mainly limited by diffusion‐controlled processes. As the scan rate increases from 0.2 to 1.0 mV s^−1^, the pseudocapacitive contribution in the HQOGP system increases from 61% to 88%, substantially exceeding that of the ZnSO_4_ system (Figure 5g,h), thereby confirming its superior reaction kinetics.

Long‐term cycling performance was further evaluated to assess the durability of Zn//VO_2_ full cells. At a high current density of 5 A g^−1^, the HQOGP‐based cell retains 87.73% of its initial capacity after 2000 cycles, significantly outperforming the cell using the pristine ZnSO_4_ electrolyte. Rate capability and galvanostatic charge–discharge (GCD) profiles (Figure 5j and Figures S19 and S20) further corroborate the enhanced electrochemical performance. Additionally, self‐discharge measurements reveal that after 50 h of open‐circuit storage, the HQOGP system maintains a capacity retention of 94.22%, markedly higher than the 71.69% observed for the ZnSO_4_ system, indicating effective suppression of parasitic reactions and charge dissipation. Overall, the introduction of HQOGP as an electrolyte additive markedly enhances electrochemical reversibility, ion transport kinetics, and long‐term capacity retention, thereby significantly improving the practical performance of Zn//VO_2_ AZIBs.

## 4 Conclusion

In summary, this study identifies arbutin, a naturally occurring glycoside, as a multifunctional electrolyte additive capable of simultaneously addressing the key degradation mechanisms in AZIBs. Through preferential interfacial adsorption and hydrogen‐bond network reconstruction, HQOGP establishes a water‐deficient interfacial environment that regulates Zn deposition behavior and suppresses water‐induced parasitic reactions. The strong affinity of HQOGP toward the Zn(101) facet promotes preferential crystallographic growth, while its hydroxyl‐rich glucose moiety reduces water activity and stabilizes the electrolyte environment. Consequently, the Zn//Cu half‐cell achieves an average Coulombic efficiency of 99.7% over 1600 cycles at 1 mA cm^−2^, and the Zn//VO_2_ full cell retains 87.73% of its initial capacity after 2000 cycles at 5 A g^−1^. These findings highlight a molecular‐level design paradigm in which a single additive simultaneously regulates the electrolyte and electrode interface, offering a promising route toward durable and high‐performance aqueous metal batteries.

## Conflicts of Interest

The authors declare no conflicts of interest.

## Supporting information




**Supporting File**: smll74607‐sup‐0001‐SuppMat.docx.

## Data Availability

The data that support the findings of this study are available from the corresponding author upon reasonable request.
