# Supplementary material for: From Bonds to Barriers: Amphiphilic Molecules Enable Dual Interfacial and Electrolyte Regulation for Highly Stable Aqueous Zinc‐Ion Batteries
Source: Small. 2026 Jul 16;22(51):e74607. doi: 10.1002/smll.74607 (PMC13568981; doi:10.1002/smll.74607)
Supplement: Supplementary file 1 [file SMLL-22-e74607-s001.docx]

**Supplementary material**

**From Bonds to Barriers: Amphiphilic Molecules Enable Dual Interfacial and Electrolyte Regulation for Highly Stable Aqueous Zinc-Ion Batteries**

Min Li^a,†^, Chao Li^b,†^, Lei Jiang^a^, Xin Tan^b,[1]*^, Kwok-Ho Lam^c,[2]*^, Qiaoji Zheng^a,[3]*^, Dunmin Lin^a^

^a^ College of Chemistry and Materials Science, Sichuan Normal University, Chengdu 610066, P. R. China

^b^ Institute for Carbon Neutralization Technology, College of Chemistry and Materials Engineering, Wenzhou University, Wenzhou 325035, Zhejiang, P. R. Chin

^c^ James Watt School of Engineering, University of Glasgow, Glasgow, G12 8QQ, United Kingdom

**Number of Pages: 21**

**Number of Figures: 20**

**Number of Tables: 1**

**1. Correlation formula**

**1.1 Electric Double-Layer Capacitance (EDLC)**

The electric double-layer capacitance (*C*) was determined from CV measurements of Zn//Zn symmetric cells and calculated using the following equation:

where *C* is the double-layer capacitance, *i*_c_ is the capacitive current, and *v* is the scan rate.

**1.2 Exchange Current Density**

The exchange current density (*I*) was calculated using the following formula：

$$I=I_{0}\frac{2F}{RT}\text{η}$$

where *I*_0_ is the measured exchange current density, *F* is the Faraday constant, *R* is the universal gas constant, *T* is the absolute temperature, and *η* is the overpotential at the nucleation point.

**1.3 Ionic Conductivity**

The ionic conductivity (*σ*) of electrolytes was calculated using the following equation:

$$\sigma=\frac{l}{RA}$$

where $l$ is the distance between the two stainless-steel electrodes, *R* is the bulk resistance of the electrolyte, and *A* is the effective contact area between the electrolyte and electrodes.

**1.4 Cation Transport Number**

The cation transport number (*t*) was determined using the Bruce–Vincent method according to the following equation:

$$\text{t}=\frac{I_{\text{s}}(\Delta V-I_{0}R_{0})}{I_{0}(\Delta V-I_{s}R_{s})}$$

where ∆*V* is the applied DC polarization voltage (25 mV), *I*_0_ and *R*_0_ are the initial current and resistance, and *I*_s_ and *R*_s_ are the steady-state current and resistance, respectively.

**1.5 Activation Energy Calculation**

The activation energy (*E*ₐ) for ion transport was determined using the Arrhenius relationship:

$$\ln(\frac{1}{R_{\text{ct}}})=\ln A-\frac{E_{a}}{RT}$$

where $\text{R}_{\text{ct}}\text{ }$is the charge-transfer resistance, $\text{A}$ is the pre-exponential factor, $\text{E}_{\text{a}}\text{ }$is the activation energy, *R* is the gas constant, *T* is the absolute temperature.

**1.6 Zn^2+^ Diffusion Coefficient**

The Zn²⁺ diffusion coefficient (*D*) was calculated from galvanostatic intermittent titration technique (GITT) measurements using the following equation:

$$D=\frac{4L^{2}}{\pi\tau}\left(\frac{\Delta E_{s}}{\Delta E_{t}}\right)^{2}$$

where *τ* is the duration of the current pulse, *L* is the Zn^2+^ diffusion length (equivalent to the thickness of the active material), ΔE_s_ and ΔE_t_ are the changes in steady-state potential after relaxation (V) and transient voltage during the current pulse (V), respectively.

**2.** **Theoretical Calculation**

### 2.1 Molecular Dynamics (MD) Simulations

#### Molecular dynamics (MD) simulations were performed using LAMMPS ^1-2^. Initial configurations were constructed using PACKMOL ^3^ and Moltemplate ^4^. A cubic simulation box with periodic boundary conditions and dimensions of 50 × 50 × 50 Å^3^ was employed.

#### For the baseline ZnSO₄ electrolyte, the system consisted of 100 ZnSO₄ units and 2000 H₂O molecules. For the HQOGP-containing electrolyte, 10 HQOGP molecules were additionally introduced into the same system. Force field parameters for the organic components were derived from the OPLS force field ^5^ using Ligpargen ^6-8^, with restrained electrostatic potential (RESP) charges assigned ^9-10^. The force field parameters for ZnSO₄ were adopted from literature [DOI:10.1063/1.5121392]. To mitigate overestimation of electrostatic interactions, the partial charges of Zn^2+^ and SO_4_^2-^ ions were uniformly scaled by a factor of 0.85, following the method proposed by Vega and Acevedo ^11-12^.

#### Water molecules were modeled using the SPC/E model. A Lennard-Jones cutoff distance of 12 Å was applied, and the long-range electrostatic interactions were treated using the particle-particle particle-mesh (PPPM) solver ^13^.

#### Energy minimization was initially performed using the conjugate gradient (CG) algorithm. Subsequently, the system was equilibrated under the isothermal-isobaric (NPT) ensemble at 300 K for 5 ns, followed by a 15 ns production run under the canonical (NVT) ensemble. Structural properties of the electrolyte, including radial distribution functions (RDFs), were analyzed based on the final 10 ns of the trajectory. Visualization and trajectory analysis were performed using VMD ^14^.

### 2.2 Density Functional Theory (DFT) Calculations

#### 2.2.1 Quantitative Calculations

Binding energy calculations were performed using the Gaussian 09 software package. All molecular structures were fully optimized at the PBE0-D3(BJ)/def2tzvp level of theory, and single-point energy calculations were conducted at the same level. The aqueous environment was simulated using the SMD implicit solvation model ^15^.

Electrostatic potential (ESP) were conducted using Multiwfn 3.8 ^10^, ^16^, and the resulting isosurfaces were visualized with VMD 1.9.3 ^14^. The binding energy of the complexes was calculated using the following equation:

$$E_{\text{binding energy}}=E_{AB}-E_{A}-E_{B}$$

where *E*_AB_, *E*_A_, and *E*_B_ denote the total energies of the complex, isolated component A, and isolated component B, respectively.

#### 2.2.2 Adsorption Energy Calculations of H_2_O and HQOGP on Zn Surfaces

Spin-polarized DFT calculations were performed using the VASP program^17-19^, which employs a plane-wave basis set in combination with the projector augmented- wave (PAW) method for core–valence electron interactions^18^. The exchange–correlation interactions were described using the generalized gradient approximation with the Perdew, Burke, and Ernzerhof functional (GGA-PBE)^20^. Long-range van der Waals (vdW) interactions were accounted for using Grimme’s DFT-D3 dispersion correction^21^.

A plane-wave energy cutoff of 400 eV was applied. Geometry optimizations were performed until the residual atomic forces were less than 0.02 eV/Å, and the total energy convergence criterion was set to 10⁻⁵ eV. Brillouin zone sampling was performed using a (2 × 2 × 1) Monkhorst-Pack k-point mesh^22^. Differential charge density distributions were visualized using VESTA^23^.

3. **Supporting Figures**

**Figure S1.** CV curves for Zn//Zn cells measured within a voltage range of −15 mV to 15 mV at various scan rates in pristine ZnSO_4_ and HQOGP-containing electrolytes.

**Figure S2.** Capacitive current as a function of scan rate for Zn//Zn cells cycled in 2 M ZnSO_4_ electrolytes with and without HQOGP.

**Figure S3.** Relative intensity ratios of Zn (101), Zn (002), and Zn (100) at different sedimentation times.

**Figure S4.** Chronoamperometric responses of Zn anode in pristine ZnSO_4_ and HQOGP-containing electrolytes measured at an overpotential of –150 mV.

**Figure S5.** Nucleation overpotential profiles of Zn//Ti cells operated in different electrolytes.

**Figure S6.** *In situ* optical microscopy images illustrating Zn deposition behavior in different electrolytes.

**Figure S7.** Polarization voltages of Zn//Zn symmetric cells in pristine ZnSO_4_ and HQOGP-containing electrolytes.

**Figure S8.** Exchange current density plots of Zn//Zn symmetric cells using pristine ZnSO_4_ and HQOGP-containing electrolyte.

**Figure S9.** Zn^2+^ transference number of the ZnSO_4_ electrolyte.

**Figure S10.** Zn^2+^ transference number of the HQOGP-containing electrolyte.

**Figure S11.** Electrochemical impedance spectra of electrolytes measured over a temperature range from 313.15 to 353.15 K.

**Figure S12.** SEM image and corresponding EDS elemental mapping of bare Zn foil after immersed in ZnSO_4_ electrolyte for 7 days.

**Figure S13.** SEM image and corresponding EDS elemental mapping of bare Zn foil after immersed in HQOGP-containing for 7 days.

**Figure S14.** XRD pattern of VO_2_.

**Figure S15.** SEM image of VO_2_.

**Figure S16.** GITT profiles of Zn//VO₂ cells in the pristine ZnSO_4_ electrolyte.

**Figure S17.** CV curves of Zn//VO_2_ full cells measured at scan rates from 0.2 to 1.0 mV s^−1^ in the pristine ZnSO_4_ electrolyte.

**Figure S18.** Corresponding log(*i*) versus log(*v*) plots derived from CV measurements at different redox peaks.

**Figure S19.** GCD profiles of Zn//VO₂ cells in the pristine ZnSO_4_ electrolyte.

**Figure S20.** GCD profiles of Zn//VO₂ cells in the HQOGP-containing electrolyte.

**Table S1** Performance comparison of reported Zn//Zn symmetrical cells using different electrolyte systems and additive strategies.

| Additive | Current density (mA cm^-2^) | Cycle life  (h) | Cumulative plating capacity (CPC) (mAh cm^-2^) | Reference | |
| --- | --- | --- | --- | --- | --- |
| **This work** | **10** | **800** | **4000** | | — |
| TXA | 1 | 2050 | 1025 | | ^24^ |
| LA | 5 | 1050 | 2625 | | ^25^ |
| LBG | 2 | 1160 | 1160 | | ^26^ |
| MBA | 1 | 1600 | 800 | | ^27^ |
| FRU | 0.5 | 5000 | 2500 | | ^28^ |
| 15-CE-5 | 10 | 152 | 760 | | ^29^ |
| CMC+SABS | 3 | 1600 | 2400 | | ^30^ |
| HPMA | 0.5 | 3500 | 875 | | ^31^ |
| DSF | 5 | 1360 | 3400 | | ^32^ |
| Cd^3+^ | 1 | 2100 | 1050 | | ^33^ |
| DTPA | 1 | 5000 | 2500 | | ^34^ |
| DCP5 | 0.5 | 4500 | 2250 | | ^35^ |
| DG | 1 | 1750 | 1750 | | ^36^ |
| CTAB | 2 | 1000 | 2000 | | ^37^ |
| IS | 1 | 1800 | 900 | | ^38^ |

**References:**

(1)	PLIMPTON, S. Fast parallel algorithms for short-range molecular dynamics. *J. Comput. Phys.* **1995**, *117*, 1-19.

(2)	Thompson, A. P.; Aktulga, H. M.; Berger, R.; Bolintineanu, D. S.; Brown, W. M.; Crozier, P. S.; In T Veld, P. J.; Kohlmeyer, A.; Moore, S. G.; Nguyen, T. D.et al. LAMMPS - a flexible simulation tool for particle-based materials modeling at the atomic, meso, and continuum scales. *Comput. Phys. Commun.* **2022**, *271*, 108171.

(3)	Martínez. L; R. ANDRADE; E. G. BIRGIN; MARTINEZ, J. M. PACKMOL: A package for building initial configurations for molecular dynamics simulations. *J. Comput. Chem.* **2009**, *30*, 2157-2164.

(4)	Jewett, A. I.; Stelter, D.; Lambert, J.; Saladi, S. M.; Roscioni, O. M.; Ricci, M.; Autin, L.; Maritan, M.; Bashusqeh, S. M.; Keyes, T.et al. Moltemplate: A Tool for Coarse-Grained Modeling of Complex Biological Matter and Soft Condensed Matter Physics. *J. Mol. Biol.* **2021**, *433*, 166841.

(5)	Jorgensen, W. L.; Maxwell, D. S.; Tirado-Rives, J. Development and Testing of the OPLS All-Atom Force Field on Conformational Energetics and Properties of Organic Liquids. *J. Am. Chem. Soc.* **1996**, *118*, 11225-11236.

(6)	Jorgensen, W. L.; Tirado-Rives, J. Potential energy functions for atomic-level simulations of water and organic and biomolecular systems. *Proc. Natl. Acad. Sci. U. S. A.* **2005**, *102*, 6665-6670.

(7)	Dodda, L. S.; Vilseck, J. Z.; Tirado-Rives, J.; Jorgensen, W. L. 1.14*CM1A-LBCC: Localized Bond-Charge Corrected CM1A Charges for Condensed-Phase Simulations. *The Journal of Physical Chemistry B* **2017**, *121*, 3864-3870.

(8)	Dodda, L. S.; Cabeza De Vaca, I.; Tirado-Rives, J.; Jorgensen, W. L. LigParGen web server: an automatic OPLS-AA parameter generator for organic ligands. *Nucleic Acids Res.* **2017**, *45*, W331-W336.

(9)	Bayly, C. I.; Cieplak, P.; Cornell, W.; Kollman, P. A. A well-behaved electrostatic potential based method using charge restraints for deriving atomic charges: the RESP model. *Journal of Physical Chemistry (1952)* **1993**, *97*, 10269-10280.

(10)	Lu, T.; Chen, F. Multiwfn: a multifunctional wavefunction analyzer. *J. Comput. Chem.* **2012**, *33*, 580-592.

(11)	Zeron, I. M.; Abascal, J. L. F.; Vega, C. A force field of Li^+^, Na^+^, K^+^, Mg^2+^, Ca^2+^, Cl^−^, and SO_4_^2−^ in aqueous solution based on the TIP4P/2005 water model and scaled charges for the ions. *The Journal of Chemical Physics* **2019**, *151*, 134504.

(12)	Doherty, B.; Zhong, X.; Gathiaka, S.; Li, B.; Acevedo, O. Revisiting OPLS Force Field Parameters for Ionic Liquid Simulations. *J. Chem. Theory Comput.* **2017**, *13*, 6131-6145.

(13)	Hockney, R. W.; Eastwood, J. W. Computer Simulation Using Particles. **2021**.

(14)	Humphrey, W.; Dalke, A.; Schulten, K. VMD: visual molecular dynamics. *Journal of Molecular Graphics* **1996**, *14*, 28-33.

(15)	Marenich, A. V.; Cramer, C. J.; Truhlar, D. G. Universal Solvation Model Based on Solute Electron Density and on a Continuum Model of the Solvent Defined by the Bulk Dielectric Constant and Atomic Surface Tensions. *The Journal of Physical Chemistry B* **2009**, *113*, 6378-6396.

(16)	Zhang, J.; Lu, T. Efficient evaluation of electrostatic potential with computerized optimized code. *Phys. Chem. Chem. Phys.* **2021**, *23*, 20323-20328.

(17)	Stephens, P. J.; Devlin, F. J.; Chabalowski, C. F.; Frisch, M. J. Ab Initio Calculation of Vibrational Absorption and Circular Dichroism Spectra Using Density Functional Force Fields. *Journal of Physical Chemistry (1952)* **1994**, *98*, 11623-11627.

(18)	Kresse, G.; Joubert, D. From ultrasoft pseudopotentials to the projector augmented-wave method. *Phys. Rev. B* **1999**, *59*, 1758-1775.

(19)	Kresse, G.; Furthmüller, J. Efficient iterative schemes for ab initio total-energy calculations using a plane-wave basis set. *Physical Review. B, Condensed Matter* **1996**, *54*, 11169-11186.

(20)	Perdew, J. P.; Burke, K.; Ernzerhof, M. Generalized Gradient Approximation Made Simple. *Phys. Rev. Lett.* **1996**, *77*, 3865-3868.

(21)	Grimme, S.; Ehrlich, S.; Goerigk, L. Effect of the damping function in dispersion corrected density functional theory. *J. Comput. Chem.* **2011**, *32*, 1456-1465.

(22)	Hendrik J. Monkhorst, J. D. P. Special points for Brillonin-zone integrations* Hendrik J. Monkhorst and James D. Pack. *Phys. Rev. B* *13*, 5188-5192.

(23)	Momma, K.; Izumi, F. VESTA 3 for three-dimensional visualization of crystal, volumetric and morphology data. *J. Appl. Crystallogr.* **2011**, *44*, 1272-1276.

(24)	Yin, J.; Liu, H.; Li, P.; Feng, X.; Wang, M.; Huang, C.; Li, M.; Su, Y.; Xiao, B.; Cheng, Y.et al. Integrated electrolyte regulation strategy: Trace trifunctional tranexamic acid additive for highly reversible Zn metal anode and stable aqueous zinc ion battery. *Energy Storage Mater.* **2023**, *59*, 102800.

(25)	Min Shi; Chengjun Lei; Huijian Wang; Pengjie Jiang; Chen Xu; Wei Yang; Xin He; Liang, X. Molecule Engineering of Sugar Derivatives as Electrolyte Additives for Deep-Reversible Zn Metal Anode. *Angewandte Chemie International Edition* **2024**, *63*, e202407261.

(26)	Wang, K.; Zhan, H.; Su, W.; Liu, X.; Sun, X. Ordered interface regulation at Zn electrodes induced by trace gum additives for high-performance aqueous batteries. *Energy Environ. Sci.* **2025**, *18*, 1398-1407.

(27)	Chen, D.; Ma, X.; Xu, W.; Yan, C.; Lyu, P.; Zhu, Q.; Yu, H.; Gao, Z.; Lv, C. Disrupting Hydrogen Bond Network Connectivity With a Double‐Site Additive for Long‐Life Aqueous Zinc Metal Batteries. *Exploration* **2025**, *5*, 20240007.

(28)	Jinliang Yan; Haozhen Dou; Mengke Su; Mi Xu; Sitong Liu; Siqi Qin; Beinuo Zhang; Kai Zong; Lichao Tan; Xin Wanget al. Molecularly Engineered Circular Additive with Multi-site Desolvation for High-performance Zinc Ion Battery. *Angewandte Chemie International Edition* **2025**, *64*, e202505372.

(29)	Xin, T.; Zhou, R.; Xu, Q.; Yuan, X.; Zheng, Z.; Li, Y.; Zhang, Q.; Liu, J. 15-Crown-5 ether as efficient electrolyte additive for performance enhancement of aqueous Zn-ion batteries. *Chem. Eng. J.* **2023**, *452*, 139572.

(30)	Li, S.; Wang, S.; Zhu, J. Advanced dual-anionic electrolyte additive for high-performance aqueous zinc-ion batteries. *Chem. Eng. J.* **2025**, *523*, 168682.

(31)	Li, J.; Long, Y.; Yu, X.; Li, J.; Li, N.; Han, J.; Wang, J.; Yang, Z. Polymeric acid additive strategy for long-lifetime aqueous zinc-ion batteries. *Energy Storage Mater.* **2025**, *76*, 104154.

(32)	Li, J.; Zhang, S.; Yu, X.; Li, J.; Gu, H.; Long, Y.; Li, N.; Han, J.; Yang, Z. Enhancing long-term cycling stability in aqueous zinc-ion batteries via effective control of bulk electrolyte with biomass-derived multifunctional additive. *Nano Energy* **2025**, *141*, 111049.

(33)	Jiang, L.; Ding, Y.; Li, L.; Tang, Y.; Zhou, P.; Lu, B.; Tian, S.; Zhou, J. Cationic Adsorption-Induced Microlevelling Effect: A Pathway to Dendrite-Free Zinc Anodes. *Nano-Micro Lett.* **2025**, *17*, 202.

(34)	Lin, S.; Zhao, W.; Guo, Y.; Yu, N.; Guo, K.; Wang, X. Highly Reversible Zinc Anode Enabled by Trifunctional Diethylenetriaminepentaacetic Acid Additive. *Acs Energy Lett.* **2024**, *9*, 4614-4623.

(35)	Liu, M.; Wang, Y.; Li, Y.; Wu, F.; Li, H.; Li, Y.; Feng, X.; Long, B.; Ni, Q.; Wu, C.et al. Dual‐Functional Interfacial Layer Enabled by Gating‐Shielding Effects for Ultra‐Stable Zn Anode. *Adv. Mater.* **2024**, *36*, 2406145.

(36)	Wang, R.; Ma, Q.; Zhang, L.; Liu, Z.; Wan, J.; Mao, J.; Li, H.; Zhang, S.; Hao, J.; Zhang, L.et al. An Aqueous Electrolyte Regulator for Highly Stable Zinc Anode Under −35 to 65 °C. *Adv. Energy Mater.* **2023**, *13*, 2302543.

(37)	Liu, Z.; Wang, R.; Gao, Y.; Zhang, S.; Wan, J.; Mao, J.; Zhang, L.; Li, H.; Hao, J.; Li, G.et al. Low‐Cost Multi‐Function Electrolyte Additive Enabling Highly Stable Interfacial Chemical Environment for Highly Reversible Aqueous Zinc Ion Batteries. *Adv. Funct. Mater.* **2023**, *33*, 2308463.

(38)	Liyang Liu; Xin-Yi Wang; Zewei Hu; Xin Wang; Qingqing Zheng; Chao Han; Jiantie Xu; Xun Xu; Hua-Kun Liu; Shi-Xueet al. Electric Double Layer Regulator Design through a Functional Group Assembly Strategy towards Long-lasting Zinc Metal Batteries. *Angewandte Chemie International Edition* *63*, e202405209.

3. ^†^The authors contributed equally to this work.

   *Corresponding author: Email: xintan@wzu.edu.cn (Xin. Tan), kwokho.lam@glasgow.ac.uk (Kwok-Ho Lam), [joyce@sicnu.edu.cn](mailto:joyce@sicnu.edu.cn) (Qiaoji Zheng) Fax: +86 28 84760802, Tel: +86 28 84760802.
